# The significance of the time interval between antecedent pregnancy and diagnosis of high-risk gestational trophoblastic tumours

**DOI:** 10.1038/sj.bjc.6603416

**Published:** 2006-10-10

**Authors:** T Powles, A Young, A Sammit, J Stebbing, D Short, M Bower, P M Savage, M J Seckl, P Schmid

**Affiliations:** 1 Charing Cross Gestational Trophoblastic Centre, London, UK

**Keywords:** gestational trophoblastic disease, interval, survival

## Abstract

It is thought that the time interval between the antecedent pregnancy and diagnosis of gestational trophoblastic tumours (GTTs) may influence the outcome of these patients. In this study, we investigate the significance of this time interval. Multivariate analysis was used to investigate if the time interval was of prognostic significance from our cohort of 241 high-risk patients with GTT. Subsequent cutpoint analysis was used to determine an optimal cutpoint for the interval covariate. The outcome of these patients was plotted according to the Kaplan–Meier method. The time interval was of prognostic significance on multivariate analysis. A period of greater than 2.8 years after pregnancy was found to be of most significance. The 5-year overall survival was 62.0% (95% CI: 47–76%) for greater than 2.8 years *vs* 94% (95% CI: 91–97%) for less than 2.8 years (*P*<0.001). Multivariate analysis showed the presence of liver metastasis and the number of metastasis was also of prognostic importance. The interval between antecedent pregnancy and diagnosis in high-risk GTT is of prognostic significance. This gives some insight into the pathogenesis of the disease.

Patients with gestational trophoblastic tumours (GTTs) are stratified into low- and high-risk groups which have different treatments and outcomes (Bagshawe, 1976; Kohorn, 2001; McNeish *et al*, 2002). This stratification system was designed to identify high-risk patients who were likely to require more intensive combination chemotherapy. A number of factors, such as plasma hCG level at presentation and the number of metastasis, are used to stratify patients into these high- and low-risk groups. Each factor scores numerical points. A score of >6 (FIGO score) or >8 (Charing Cross score) defines high-risk disease.

One of these factors is the interval between the antecedent pregnancy and treatment, which if greater than 1 year scores 4 or 6 points in the FIGO or Charing Cross systems, respectively. The reason for selecting the time interval of 1 year is unclear, although it appears that tumour age in GTT is of prognostic significance (Kim *et al*, 1998). Therefore, in this study, we investigate the relationship between this time interval and outcome more closely.

## PATIENTS AND METHODS

### Data collection

The Charing Cross GTT database was screened to identify all women diagnosed between 1984 and 2004. At presentation for chemotherapy, patients were stratified into low- or high-risk groups according to their Charing Cross score and treated accordingly (Bagshawe, 1976; Kohorn, 2001; McNeish *et al*, 2002). Those patients scoring 9 or more were classed as high-risk GTT and included in this study. These patients were treated in similar manner using regimens according to protocol (Rustin *et al*, 1989; Newlands *et al*, 1998). Patients with placental site tumour were excluded because the treatment and outcomes for these patients is different (Papadopoulos *et al*, 2002).

This study was approved by our local Institutional Review Board.

### Statistical analysis

The interval between pregnancy and diagnosis was plotted as a non-linear covariate in a univariate Cox regression using natural splines. The resulting curve showed an increasing hazard ratio with respect to interval. Cutpoint analysis was used to determine an optimal cutpoint for the interval covariate. As the determination of a cutpoint may be unstable with respect to perturbations of the data, this analysis was confirmed using non-parametric bootstrapping, by repeating the original analysis 2000 times using samples drawn from the original distribution with replacement.

Patients overall survival and disease-free survival was recorded and plotted according to the Kaplan–Meier method.

Univariate and multivariate analysis was performed to identify prognostic factors associated with outcome for patients with high-risk disease. This was performed for high-risk patients using initially the Charing cross scoring system and was repeated using the FIGO scoring system to identify high-risk patients.

## RESULTS

The characteristics, treatment and outcome of the 241 patients are shown in Table 1. In this study, all patients with a time period of greater than 2.8 year group had previously histologically confirmed GTT.

**Figure 1:**
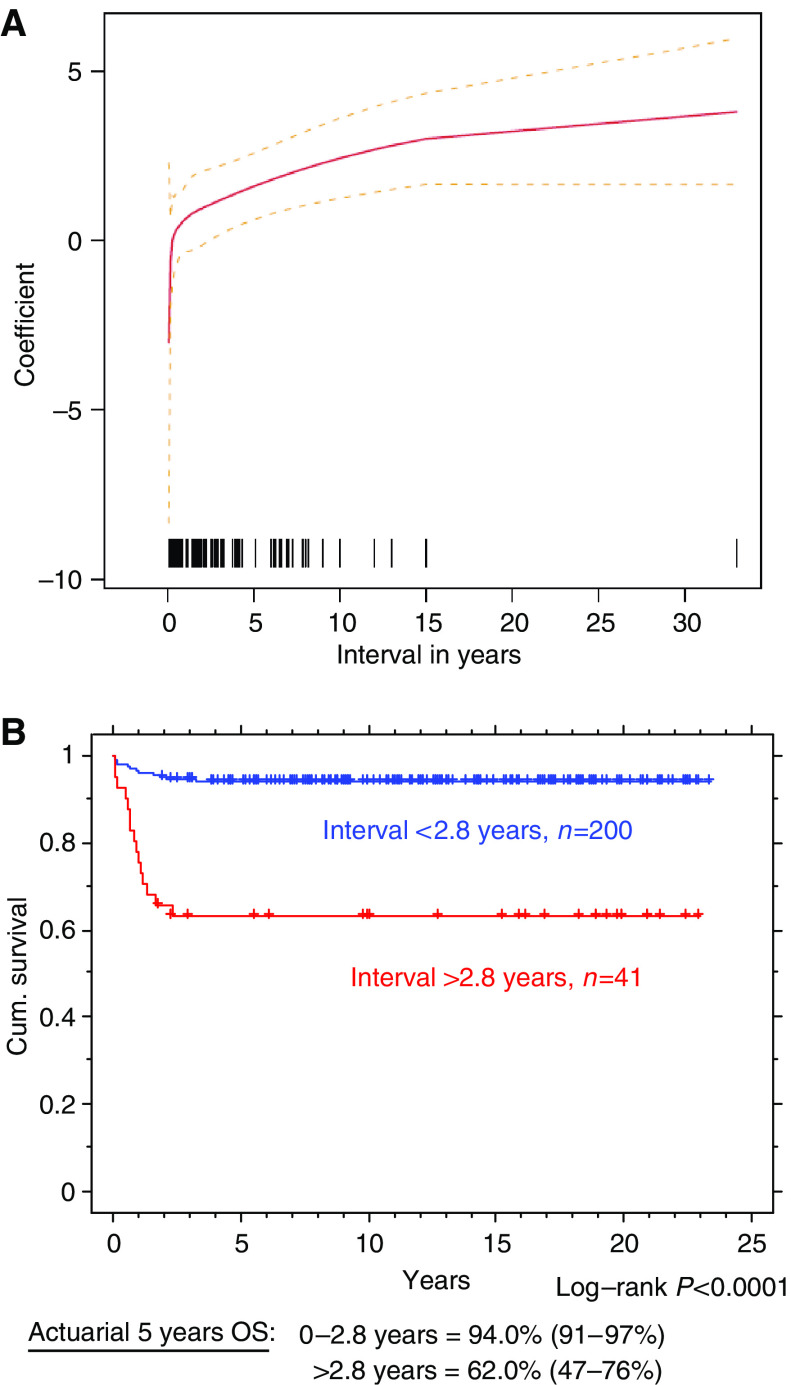
(**A**) Cutpoint analysis investigating the most significant time point between pregnancy and diagnosis. (**A**) Interval *vs* coefficient for that interval, used to derive the interval cutoff limit. Dotted lines show 95% confidence intervals. Lower rug plot shows number of measurements at each interval. (**B**) Comparison of patients with a time period of less and more than 2.8 years since the antecedent pregnancy. Log rank *P*<0.0001. Actuarial 5-year OS: 0–2.8 years=94.0% (91–97%) >2.8 years=62.0% (47–76%).

Cox regression univariate and multivariate analysis demonstrated that the time interval between pregnancy and treatment for GTT was of prognostic significance. This was true for high-risk patients identified by either the WHO or FIGO scoring systems (*P*<0.001 for both). The resulting hazard ratio from this data was non-linear and rose with increased interval. Cutpoint analysis with bootstrapping suggested that 2.8 years was the optimum cutoff point (Figure 1a). The patients were subsequently separated into two groups according to this time point. The characteristics and outcome of patients in these two groups are compared in Table 1 and Figure 1b. Patients with an interval of greater than 2.8 years had a significantly worse outcome (Table 1).

We subsequently went on to investigate the outcome of patients with a time interval <1 and 1–2.8 years, this was because 1 year is the current cutoff time point used for both scoring systems (Kim *et al*, 1998). Results showed that this group of patients has a similar outcome to patients with a time interval of less than 1 year (5-year survival 94.4% (87.3–100% *vs* 93.8% (95% CI 90–97%), respectively), but a better outcome compared to patients with a time interval of greater than 2.8 years (62.0% (95% CI 47–76%): *P*<0.01).

Multivariate analysis showed three prognostic factors, which independently predict the outcome of patients with high-risk disease. These include an increased time interval between antecedent pregnancy and diagnosis of high-risk disease (as described earlier), the presence of liver metastasis at diagnosis and more than eight metastasis at diagnosis. Other factors such as hCG at presentation, age and the presence of lung metastasis were not significant in multivariate analysis. Other possible confounding factors such as a term pregnancy or ancedental pregnancy were not found to be significant in multivariate analysis. Both the WHO and FIGO scoring systems were significant for prognosis in using univariate analysis. These scoring systems were precluded from the multivariate analysis as they took into account a number of potentially significant independent univariate factors.

## DISCUSSION

These data show that an increased time interval between pregnancy and diagnosis of GTT is associated with a worse outcome. This appears particularly marked after 2.8 years.

The reason for excess mortality as the time interval increases is unknown. Possible confounding factors are that patients with a greater time interval may have more advanced disease at presentation or be more likely to initially have a term pregnancy (both associated with a worse prognosis). However, multivariate analysis confirmed the time interval as an independent prognostic factor.

Alternatively, these findings may occur because a longer interval may be a consequence of slower tumour growth rates, allowing an increase in the development of mutations, which are associated with chemotherapy resistance in other tumour types (Kelland *et al*, 1992). Gestational trophoblastic tumour is the only cancer where the date of the occurrence of the tumour is truly known, and this extended period between occurrence and diagnosis being associated with a poor outcome gives us some insight into the pathogenesis this and perhaps other cancers.

Unlike previous studies of high risk GTT, these data exclude all patients with placental site tumours, which has different disease characteristics and should be considered as a separate disease process (Papadopoulos *et al*, 2002). The exclusion of these patients may account for the relatively good overall survival in this series, compared with other published high-risk disease series (Vaeth *et al*, 1984; Bower *et al*, 1997). It also allows us, for the first time, to specifically investigate for prognostic factors in these high-risk patients. Multivariate analysis showed that time interval between pregnancy and diagnosis, liver metastasis and greater than eight metastasis at diagnosis were associated with poor prognosis. It is noteworthy that hCG at presentation, which is an important factor in separation between low- and high-risk disease, is of no prognostic importance in high-risk disease.

In summary, our findings suggest an increased time interval pregnancy and diagnosis of GTT is of prognostic importance and this gives us some insight into the pathogenesis of the disease. It appears that a cut of 2.8 years is most significant. In view of the poorer outcome of these patients, perhaps they should be considered for more aggressive initial treatment.

## Figures and Tables

**Table 1:** Patient characteristics

	**All patients**	**Less than 2.8 years since pregnancy**	**Greater than 2.8 years since pregnancy**
Number	241	200	41
Median age	30 (range 17–61)	33 (range 17–54)	35 (range 22–61)
Median Prognostic score (WHO).	11 (range 8–32)	11 (range 8–32)	14 (range 9–32)
Median Prognostic score (FIGO).	8 (range 4–20)	8 (range 4–18)	9 (range 5–20)
Median follow-up	11.7 years	11.9 years	6.2 years
	Range 0.1–23.8	Range 0.9–23.8	Range 0.1–23.8
Median HCG at presentation	190 000	200 000	72 000
	300–3.4 million	300–3.3million	450–3.4million
			
*Chemotherapy regimens*			
EMA/CO	206 (85%)	174 (87%)	32 (80%)
EP/EMA	14 (6%)	9 (5%)	5 (12%)
EMA/CNS	17 (7%)	14 (7%)	3 (7%)
Other	4 (1%)	3 (2%)	1 (1%)
			
*Causal pregnancy*			
Term	125 (52%)	96 (48%)	29 (70%)
Abortion/unknown	37 (15%)	32 (16%)	6 (15%)
Molar	79 (33%)	73 (35%)	6 (15%)
			
*Sites of metastasis*			
Pulmonary	98 (41%)	77 (39%)	21 (51%)
Extrapulmonary	38 (16%)	30 (15%)	8 (20%)
Both	26 (10%)	18 (9%)	8 (20%)
			
Deaths	27 (11%)	12 (6%)	15 (37%)
Cancer-related deaths	23 (10%)	10 (5%)	13 (32%)
5-year survival	87.4%	94.0%	62.0%
	95% CI: (77.8–96.8)	95% CI: (91.3–97.0%)	(95% CI: 47.2–76.8%)

CI=confidence interval; CNS=central nervous system; EMA=epithelial membrane antigen; FIGO=International Federation of Gynecologic and Obstetrics; WHO=World Health Organization.
