# The significance of the time interval between antecedent pregnancy and diagnosis of high-risk gestational trophoblastic tumours

**DOI:** 10.1038/sj.bjc.6603536

**Published:** 2006-12-12

**Authors:** T Powles, A Young, A Sanitt, J Stebbing, D Short, M Bower, P M Savage, M J Seckl, P Schmid

**Correction to**: *British Journal of Cancer* (2006) **95**, 1145–1147, doi: 10.1038/sj.bjc.6603416

Owing to an error on the author's part, the name of the third author was incorrectly spelt as ‘Sammit’. The correct spelling is now shown above.

